# Two‐Wave Variable Nanotheranostic Agents for Dual‐Mode Imaging‐Guided Photo‐Induced Triple‐Therapy for Cancer

**DOI:** 10.1002/advs.202201834

**Published:** 2022-08-02

**Authors:** Xiao Sang, Tong Gao, Xiaoqing Liu, Yelong Shen, Lili Chang, Shunli Fu, Han Yang, Huizhen Yang, Weiwei Mu, Shuang Liang, Zipeng Zhang, Na Zhang, Yongjun Liu

**Affiliations:** ^1^ Department of Pharmaceutics Key Laboratory of Chemical Biology (Ministry of Education) NMPA Key Laboratory for Technology Research and Evaluation of Drug Products School of Pharmaceutical Sciences Cheeloo College of Medicine Shandong University 44 Wenhua Xi Road Jinan Shandong Province 250012 China; ^2^ Department of Radiology Shandong Provincial Hospital Affiliated to Shandong First Medical University 324 Jingwu Weiqi Road Jinan Shandong Province 250021 China

**Keywords:** antitumor immune response, dual‐mode imaging, imaging‐guided triple‐therapy, magnetic resonance imaging, nanotheranostics, near‐infrared fluorescence imaging, photothermal therapy

## Abstract

Photothermal therapy (PTT) is a promising strategy for cancer treatment, but its clinical application relies heavily on accurate tumor positioning and effective combination. Nanotheranostics has shown superior application in precise tumor positioning and treatment, bringing potential opportunities for developing novel PTT‐based therapies. Here, a nanotheranostic agent is proposed to enhance magnetic resonance imaging (MRI)/ near‐infrared fluorescence imaging (NIRFI) imaging‐guided photo‐induced triple‐therapy for cancer. Thermosensitive liposomes co‐loaded with SPIONs/IR780 and Abemaciclib (SIA‐TSLs), peptide ACKFRGD, and click group 2‐cyano‐6‐amino‐benzothiazole (CABT) are co‐modified on the surface of SIA‐TSLs to form SIA‐*α*TSLs. ACKFRGD can be hydrolyzed to expose the 1, 2‐thiolamino groups in the presence of cathepsin B in tumors, which click cycloaddition with the cyano group on CABT, resulting in the formation of SIA‐*α*TSLs aggregates. The aggregation of SIA‐*α*TSLs in tumors enhances the MRI/NIRFI imaging capability and enables precise PTT. Photo‐induced triple‐therapy enhances precision cancer therapy. First, PTT ablates specific tumors and induces ICD via localized photothermal. Second, local tumor heating promotes the rupture of SIA‐*α*TSLs, which release Abemaciclib to block the tumor cell cycle and inhibit Tregs proliferation. Third, injecting GM‐CSF into tumor tissue leads to recruitment of dendritic cells and initiation of antitumor immunity. Collectively, these results present a promising nanotheranostic strategy for future cancer therapy.

## Graphical abstract

A two‐wave variable nanotheranostics strategy is proposed to enhance magnetic resonance imaging/near‐infrared fluorescence imaging dual‐mode imaging‐guided photo‐induced triple‐therapy for cancer. The nanotheranostic agents SIA‐*α*TSLs possess the ability of precise localization and light‐controlled drug release, which is beneficial to improve chemo/photo/immuno‐therapeutic efficiency. In general, this unique programmed variable nanotheranostics strategy is expected to achieve precise image‐guided tumor localization therapy.

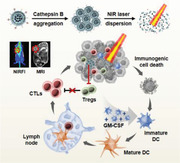

## 1 Introduction

Photothermal therapy (PTT) is a promising strategy for local thermal tumor ablation. Lasers are used to irradiate tumor sites by exploiting their high energy to induce heating, thus resulting in cell damage.^[^ ^1^ ^]^ For example, Nd:YAG lasers have been used clinically for endoscopic irradiation of obstructing endobronchial cancers.**^[^** ^2^ ^]^ After PTT, the damaged cells release damage‐associated molecular patterns (DAMPs) such as adenosine triphosphate (ATP), high mobility group protein B1 (HMGB1), and calreticulin (CRT), which act as immunogenic signals that promote dendritic cells (DCs) to engulf tumor cells and activate the host's immune system to fight tumors.^[^ ^3^ ^]^ To further improve the effect of PTT, contrast‐enhanced PTT using near‐infrared (NIR) photosensitizers has been employed, which offers the advantages of lower power, better selectivity, and superior therapeutic effects.**^[^** ^4^ ^]^ Despite these promises, accurate tumor positioning for laser irradiation is a prerequisite for effective PTT, since the technique suffers from challenges in the positioning of the tumor location during the treatment process. Otherwise, the residual or tumor metastasis tumor cells outside the laser irradiation range lead to certain limitations to the usage of PTT alone in tumor treatment.^[^ ^5^ ^]^

Nanotheranostics serves as an effective approach to ensure precise localization of laser irradiation, and has been developed to achieve diverse imaging and therapeutic strategies.^[^ ^6^ ^]^ Nanotheranostics agents integrate imaging agents and therapeutic drugs into one space, and target accumulation in tumor tissues, which is expected to enhance the imaging capabilities and achieve optimal therapeutic effects.^[^ ^7^ ^]^ Combining PTT with nanotheranostics is a promising strategy for the precise localization of laser irradiation. Precise directional guidance and real‐time monitoring could provide key information for the local thermal ablation of tumors.^[^ ^8^ ^]^ Thus, the development of PTT‐based nanotheranostics is of great significance for accurate PTT tumor positioning, and has an important impact on PTT‐based clinical applications. In clinical, magnetic resonance imaging (MRI), computed tomography, positron emission tomography, NIR fluorescence imaging (NIRFI), and other imaging modalities have been used for the localization and auxiliary diagnosis of various solid malignant tumors.^[^ ^9^ ^]^ At present, multimodal imaging has been widely used in research and clinical applications to enhance the accuracy of medical diagnosis, thus enabling precise and reliable information at disease sites.^[^ ^10^ ^]^ The MRI/NIRFI dual‐mode system realizes the complementarity of the diagnostic parameters, which significantly improves the quantification accuracy and spatial resolution of tumor positioning.^[^ ^11^ ^]^

**Scheme 1:**
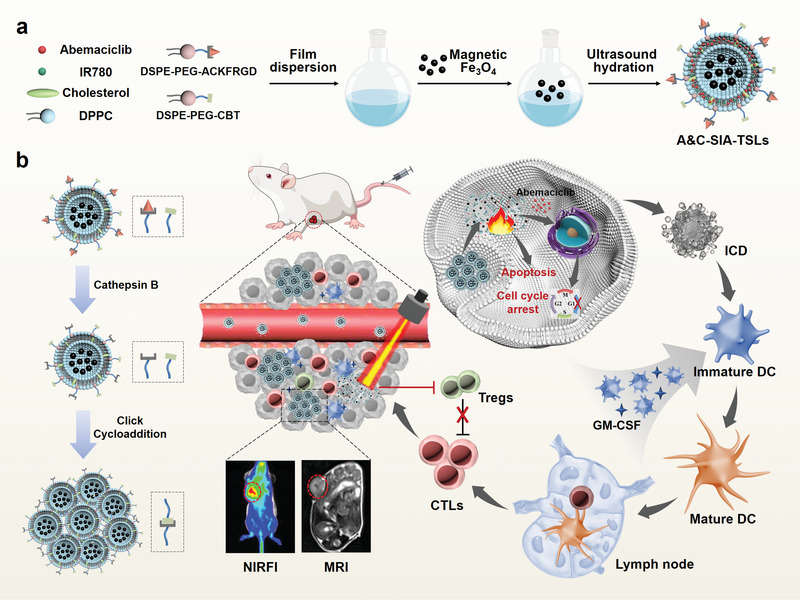
Schematic illustration of the two‐wave variable nanotheranostics strategy for dual‐mode imaging‐guided photo‐induced triple‐therapy. a) Preparation of SIA‐*α*TSLs. b) SIA‐*α*TSLs displayed the ability of cathepsin B‐responsive aggregation and near‐infrared laser‐responsive dispersion, thus enhancing the MRI/NIRFI imaging and therapeutic effects.

In the present study, we propose a two‐wave variable nanotheranostics strategy to enhance the MRI/NIRFI dual‐mode image for precise PTT (**Scheme** **1**). The nanotheranostics agents were composed of thermosensitive liposomes (TSLs) co‐loaded with SPIONs, IR780, and the chemotherapeutic drug Abemaciclib (SIA‐TSLs). SPIONs and IR780 acted as imaging agents for MRI and NIRFI, respectively. The peptide ACKFRGD and the click group 2‐cyano‐6‐amino‐benzothiazole (CABT) were co‐modified on the surface of SIA‐TSLs to form SIA‐*α*TSLs. The SIA‐*α*TSLs could achieve the following functions: i) precisely localized light irradiation: the RGD moiety in the ACKFRGD peptide can target the tumor tissue.^[^ ^12^ ^]^ Under the action of highly expressed cathepsin B in the tumor tissue, the KFRGD moiety in ACFKRGD is cleaved to expose the AC. The 1,2‐thiolamino groups on AC undergo click cycloaddition with the contiguous cyano groups on CABT, leading to the aggregation of SIA‐*α*TSLs.^[^ ^13^ ^]^ The aggregation of SIA‐*α*TSLs in tumors enhances the MRI/NIRFI imaging capability and enables precise localized light irradiation. ii) Light‐controlled drug release: under precisely localized laser irradiation, the photosensitizer IR780/SPIONs converts light energy into heat energy, causing local hyperthermia in tumors. The local tumor temperature is higher than the phase transition temperature of TSLs, resulting in enhanced lipid membrane fluidity and permeability.^[^ ^14^ ^]^ Subsequently, morphological changes in the TSLs facilitate the rapid release of therapeutic drugs. This unique programmed variable nanotheranostics strategy is expected to achieve precise image‐guided tumor localization therapy.

Eradicating tumors completely by PTT alone is not sufficient, and thus multimodal therapy, in combination with PTT, is promising for cancer treatment. Several proposed combination partners for PTT, such as chemotherapy and immunotherapy, have already been well established.^[^ ^15^ ^]^ PTT ablates specific tumors and induces immunogenic cell death (ICD) by localized PTT. To enhance the effect of PTT, chemotherapy can not only enhance the local tumor treatment effect, but also eliminate the residual tumor cells. Meanwhile, immunotherapy can enhance the PTT‐induced ICD effect and further exert systemic antitumor immunity.^[^ ^16^ ^]^ Therefore, we propose photo‐induced photo/chemo/immunotherapy guided by dual‐mode imaging to enhance the efficiency of cancer treatment. i) After dual‐mode imaging‐guided NIR laser irradiation, local heating caused by SPIONs/IR780 promotes ICD, accompanied by the release of tumor‐associated antigens and DAMPs. ii) Local heating promotes the release of Abemaciclib, which induces G1 cycle arrest in tumor cells by inhibiting the phosphorylation of retinoblastoma.^[^ ^17^ ^]^ Abemaciclib could also effectively reduce the proportion of Tregs and secretion of IL‐10 and TGF‐*β* in tumor tissues, thus alleviating the immunosuppressive tumor microenvironment.^[^ ^18^ ^]^ iii) DCs are the most effective antigen‐presenting cells, which can uptake, process, and present antigens to trigger an effective antitumor T cell response.^[^ ^19^ ^]^ To enhance the PTT‐induced immune response, granulocyte‐macrophage colony‐stimulating factor (GM‐CSF) was injected intratumorally to recruit DCs and initiate antitumor immunity.^[^ ^20^ ^]^ Therefore, photo‐induced triple‐therapy guided by dual‐mode imaging is an effective strategy, that would greatly enhance the effect of PTT in cancer treatment.

In this study, SIA‐*α*TSLs were successfully prepared. The cathepsin B‐responsive aggregation ability was determined and the NIR laser‐responsive dispersion ability was characterized. Tumor accumulation, MRI, and NIRFI were verified in vivo and in vitro. After laser irradiation, tumor cell apoptosis and cycle arrest were detected, and the release of DAMPs (including CRT, HMGB1, and ATP) was measured to evaluate the effect of ICD. After intratumoral injection of GM‐CSF, the infiltration of DCs into the tumor tissue and the maturation of DCs in the lymph nodes were evaluated. The remission of the immunosuppressed TME was assessed by analyzing the infiltration of T cells, Tregs, cytotoxic T lymphocytes (CTLs), as well as cytokine secretion in tumors. The antitumor efficiency of dual‐mode imaging‐guided photo‐induced triple‐therapy was also investigated in CT26 tumor‐bearing mice. We believe that our study provides a rational design to achieve effective nanotheranostics, which in turn provides hope for accurate localization and efficient treatment of cancer.

## 2 Results and Discussion

### 2.1 Preparation and Characterization of SIA‐*α*TSLs

**Figure 1:**
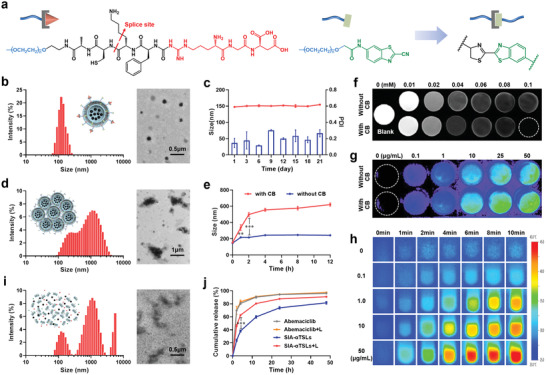
SIA‐*α*TSLs had the ability to aggregate for MRI/NIRFI imaging and rupture for drug release. a) Schematic illustration of the cathepsin B‐responsive cross‐linking of functional lipids. b) Hydrodynamic size and TEM images of SIA‐*α*TSLs. c) Stability of SIA‐*α*TSLs at 4 °C for 21 d. d) Hydrodynamic size and TEM images of the SIA‐*α*TSLs aggregates. e) Size changes of SIA‐*α*TSLs incubated with or without cathepsin B. f) T2‐weighted MRI images and g) NIRFI images of SIA‐*α*TSLs incubated with or without cathepsin B. h) Infrared thermal images of SIA‐*α*TSLs within 10 min of laser irradiation at the powder of 1.0 W cm^−2^. i) Hydrodynamic size and TEM images of SIA‐*α*TSLs aggregates after laser irradiation. j) Release profiles of Abemaciclib from SIA‐*α*TSLs with and without laser irradiation. Data were shown as mean ± SD (n  = 3). **p < 0.01 and ***p < 0.001.

The MRI contrast agents SPIONs were synthesized by thermal decomposition.^[^ ^21^ ^]^ Transmission electron microscopy (TEM) images showed that SPIONs were well‐dispersed nanoparticles with a size of 13.63 ± 1.63 nm (Figure S1, Supporting Information). Cathepsin B‐responsive cross‐linked functional lipids were obtained by modifying the cross‐linking groups on the lipid material.^[^ ^22^ ^]^ The peptide ACKFRGD and the CABT group were connected to DSPE‐PEG_2000_‐NHS through an amide reaction. DSPE‐PEG_2000_‐ACKFRGD and DSPE‐PEG_2000_‐CABT were characterized using ^1^H nuclear magnetic resonance (^1^H NMR) (Figure S2, Supporting Information). DSPE‐PEG_2000_‐ACKFRGD exhibited characteristic peaks of ACKFRGD at 6.50–8.69 ppm, while PEG exhibited characteristic peaks at 3.25–3.75 ppm, which demonstrated the successful synthesis of DSPE‐PEG_2000_‐ACKFRGD. The NH_2_ of CABT had a characteristic peak at 6.11 ppm (s, 2H), after CABT was amidated with DSPE‐PEG_2000_‐NHS, the proton peak of NH_2_ moved to 6.57 ppm (s, 1H), and DSPE‐PEG_2000_‐CABT showed a new peak at 3.25–3.75 ppm, indicating the successful synthesis of DSPE‐PEG_2000_‐CABT.^[^ ^23^ ^]^ DSPE‐PEG_2000_‐ACKFRGD could be hydrolyzed in tumors highly expressing cathepsin B to expose the AC fragment. The click cycloaddition reaction between the AC fragment and the CABT group promoted the cross‐linking of lipids at the tumor site (**Figure** **1**a).

Based on the above functional materials, SIA‐*α*TSLs were prepared using the film‐dispersion method. The modification rate of ACKFRGD and CABT was 1.64 ± 0.12% and 1.64 ± 0.02%. TEM images showed that SIA‐*α*TSLs were well‐dispersed nanoparticles with a size of 148.13 ± 4.24 nm (Figure 1b, Table S1, Supporting Information). The drug loading efficiency of Abemaciclib, IR780, and SPIONs were 9.87 ± 0.22%, 0.90 ± 0.04%, and 0.80 ± 0.05%, respectively (Table S2, Supporting Information). The ultraviolet‐visible spectrophotometry (UV–vis) absorption spectra and fluorescence spectra of the SIA‐*α*TSLs were measured (Figure S3, Supporting Information). The zeta potential of the SIA‐*α*TSLs was −4.69 ± 0.17 mV. Upon assessing the storage stability of the SIA‐*α*TSLs at 4 ℃ for 21 d, it was found that there was no obvious change in particle size (*p* > 0.05) or PDI (*p* > 0.05) (Figure 1c).

In the next step of the study, the aggregation ability of SIA‐*α*TSLs with cathepsin B was investigated. The TEM image shows that the click cycloaddition reaction triggered by cathepsin B caused aggregation of SIA‐*α*TSLs (Figure 1d). And the aggregation of SIA‐*α*TSLs at different time points after incubation with cathepsin B was observed. The results showed that the particle size of SIA‐*α*TSLs increased to 498.70 ± 23.89 nm within 2 h at pH 6.5, while the particle size of SIA‐TSLs did not change significantly (Figure 1e, Figure S4, Supporting Information). The particle size of SIA‐*α*TSLs increased to 288.12 ± 23.66 nm at pH 7.4, which may be caused by the decreased activity of cathepsin B at pH 7.4. Further, GSH or H_2_O_2_ was added to the system, and the results showed that the presence of GSH or H_2_O_2_ had no effect on the aggregation ability of SIA‐*α*TSLs (Figure S5, Supporting Information).

The SPIONs and IR780 loaded in SIA‐*α*TSLs could be used as imaging contrast agents for MRI and NIRFI respectively. To explore the influence of aggregation on imaging, we performed magnetic resonance scanning and NIR fluorescence scanning with or without cathepsin B. The results of the MRI imaging showed that the negative contrast ability of SIA‐*α*TSLs was enhanced in the presence of cathepsin B, with the signal intensity significantly lower than that without cathepsin B (Figure 1f). The transverse relaxation rate (r_2_) under different Fe concentrations was analyzed, it was 75.30 mm ^−1^s^−1^ without cathepsin B, which increased to 103.20 mm ^−1^s^−1^ with cathepsin B, indicating that cathepsin B‐mediated aggregation could enhance the T2‐weighted MRI signal (Figure S6, Supporting Information). The average fluorescence intensity of the fluorescence images had no significant difference between the groups with CB and without CB in vitro (*p* > 0.05) (Figure 1g, Figure S7, Supporting Information).

Subsequently, the rupture of SIA‐*α*TSLs under laser irradiation was verified. The temperature change under 808 nm laser irradiation was used to evaluate the in vitro photothermal conversion efficiency of SIA‐*α*TSLs. At the powers of 0.1, 0.6, 1.0, and 2.0 W cm^−2^, the temperature of SIA‐*α*TSLs increased to 28.33 ± 0.67, 40.10 ± 0.53, 44.30 ± 0.46, and 50.67 ± 0.50 ℃ respectively within 10 min (Figure S8, Supporting Information). The photothermal effects of different concentrations of SIA‐*α*TSLs were then measured. At a power of 1.0 W cm^−2^, the temperature of SIA‐*α*TSLs gradually increased within 10 min and showed a concentration‐dependent pattern (Figure 1h, Figure S9, Supporting Information). After 808 nm irradiation, rupture of the SIA‐*α*TSLs composed of the temperature‐sensitive lipids DPPC was clearly observed in the TEM image and in terms of particle size change (Figure 1i). The rupture of SIA‐*α*TSLs could promote the release of the chemotherapeutic drug Abemaciclib. The in vitro release profile of Abemaciclib from SIA‐*α*TSLs significantly increased after laser irradiation (Figure 1j, Figure S10, Supporting Information). The results showed that 46.50 ± 1.94% of Abemaciclib were released after 2h in laser‐irradiated group, with the efficiency being 1.94‐fold higher than that without laser (*p* < 0.001). These results indicated that laser irradiation increases the temperature of SIA‐*α*TSLs, thus promoting their rupture and drug release.

### 2.2 Tumor Accumulation and MRI/NIRFI Imaging of SIA‐*α*TSLs

**Figure 2:**
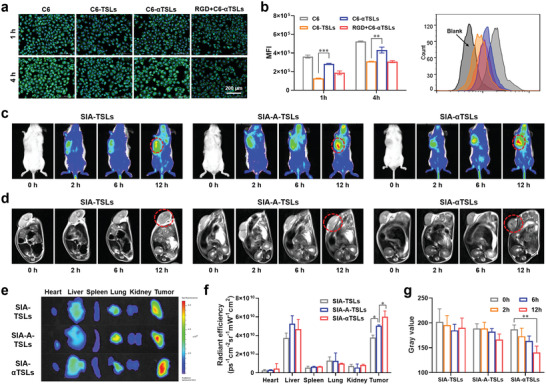
SIA‐*α*TSLs actively targeted the tumor site to achieve MRI/NIRFI dual‐mode imaging. a) Fluorescence microscopy images and b) flow cytometric analysis of cellular uptake in HUVECs. c) NIRFI images and d) T2‐weighted MRI images of SIA‐TSLs, SIA‐A‐TSLs (without CABT group), and SIA‐*α*TSLs in CT26‐tumor‐bearing mice at different time‐points. e) Ex vivo NIRFI images and f) relative fluorescence intensity of main organs and tumors after mice were sacrificed at 12 h. g) Gray values of SIA‐TSLs, SIA‐A‐TSLs, and SIA‐*α*TSLs in tumor tissues at different time‐points. Data were shown as mean ± SD (n  = 3). **p < 0.01 and ***p < 0.001.

Tumor neovascular endothelial cells and tumor cells specifically express integrin receptors, such as *α*v*β*3 and *α*v*β*5.^[^ ^24^ ^]^ RGD can specifically interact with overexpressed integrin receptors to mediate the active targeted delivery of SIA‐*α*TSLs to tumor tissues.^[^ ^25^ ^]^ Cellular uptake experiments in human umbilical vein endothelial cells (HUVECs) were performed to evaluate the active targeting ability of SIA‐*α*TSLs. Coumarin 6 (C6) was loaded into the *α*TSLs for tracking (C6‐*α*TSLs). In fluorescence microscopy images (**Figure** **2**a) and flow cytometric analysis (Figure 2b), the uptake ratio of C6‐*α*TSL in HUVECs was significantly higher than that of C6‐TSLs at 1 and 4 h (*p* < 0.001, *p* < 0.001). The cellular uptake of C6‐*α*TSL was suppressed upon pre‐treatment of HUVECs with free RGD. These results suggested that the RGD modification may mediate the active targeting of SIA‐*α*TSLs.

Encouraged by the ideal MRI and NIRFI imaging properties of SIA‐*α*TSLs in vitro, the dual‐mode positioning capability of SIA‐*α*TSLs was further evaluated in vivo. Different groups of tumor‐bearing mice were intravenously injected with SIA‐TSLs, SIA‐A‐TSLs (without CABT), or SIA‐*α*TSLs. First, the distribution of SIA‐*α*TSLs was evaluated in vivo using NIRFI (Figure 2c). Real‐time imaging showed that SIA‐*α*TSLs gradually accumulated at the tumor site after intravenous injection (Figure 2e, f). Compared to that of SIA‐TSLs, there was a significant improvement in the fluorescence signal intensities of SIA‐A‐TSLs and SIA‐*α*TSLs in tumors (*p* < 0.05, *p* < 0.001), indicating that RGD mediated active targeting of tumor accumulation. The intensity of the fluorescence signal in tumors was higher in SIA‐*α*TSLs group than that in SIA‐A‐TSLs group (*p* < 0.05), indicating that cathepsin B‐responsive aggregation enhanced the NIRFI signal. T2 imaging of each mouse was performed using an MRI scanner (Figure 2d, g). With accumulation of the formulation in the tumor tissue, the T2 signal increased gradually. At 12h after intravenous injection, the T2 signal of the tumor in SIA‐*α*TSLs group was significantly better than that in SIA‐TSLs and SIA‐A‐TSLs groups (*p* < 0.01, *p* < 0.001). The quantitative results of the signal intensity showed that the T2 signal intensity decreased by 24.81% in SIA‐*α*TSLs group, indicating that the cathepsin B‐mediated aggregation could enhance the T2‐weighted MRI signal. These results indicated that SIA‐*α*TSLs as dual‐mode imaging contrast agents could increase tumor targeting and aggregation, thereby enhancing the MRI/NIRFI signals in tumor areas.

### 2.3 Chemo‐Photothermal Therapy of SIA‐*α*TSLs

**Figure 3:**
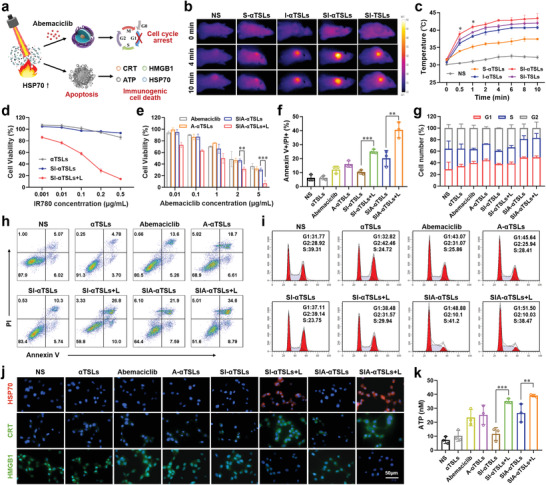
SIA‐*α*TSLs induced chemotherapy‐PTT and promoted the immunogenic death of tumor cells. a) Schematic diagram of cell cycle arrest and cell apoptosis caused by SIA‐*α*TSLs. b) Infrared thermal images and c) temperature profile of tumors within 10 min under 1.0 W cm^−2^ laser irradiation. d) Cell viability of CT26 cells upon treatment with *α*TSLs, SI‐*α*TSLs, or SI‐*α*TSLs+L. e) Cell viability of CT26 cells upon treatment with Abemaciclib, A‐*α*TSLs, SIA‐*α*TSLs, or SIA‐*α*TSLs+L. f,h) Cell apoptosis in different treated CT26 cells. g,i) Cell cycle arrest in different treated CT26 cells. j) Fluorescence microscopy images of HSP70, CRT, and HMGB1 distributions in CT26 cells. k) ATP secretion in different treated CT26 cells. Data were shown as mean ± SD (n = 3). **p < 0.01 and ***p < 0.001.

With the guidance of MRI and NIFR dual‐mode positioning, the tumor tissue was irradiated with a NIR laser to evaluate the in vivo photothermal conversion performance of SIA‐*α*TSLs. Temperatures of 42 – 45 °C have been reported to induce tumor cell apoptosis and necrosis.^[^ ^26^ ^]^ We first investigated the heating trend of tumor tissue at different powers (0.6, 1.0, and 2.0 W cm^−2^). At the powers of 0.6, 1.0, and 2.0 W cm^−2^, the temperature of the local tumor tissue increased to 37.23 ± 0.40, 43.40 ± 1.31, and 49.37 ± 0.61 °C within 10 min respectively (**Figure** **3**b, c, Figure S9, Supporting Information). It was shown that SIA‐*α*TSLs effectively enhanced the photothermal conversion efficiency of local tumor hyperthermia. Considering the damage to mouse skin induced by a higher power, a power of 1.0 W cm^−2^ was used in vivo. With laser irradiation at the power of 1.0 W cm^−2^, the temperature of the local tumor tissue in NS, S‐*α*TSLs, I‐*α*TSLs, SI‐*α*TSLs, and SI‐TSLs groups increased to 32.20 ± 0.46, 37.43 ± 0.25, 40.73 ± 0.38, 43.40 ± 1.31, and 42.03 ± 0.93 °C, respectively (Figure 3b, c).

Since local hyperthermia caused by laser irradiation could promote tumor cell death, the phototoxicity of SIA‐*α*TSLs was investigated first (Figure 3d). After treatment with SI‐*α*TSLs and laser irradiation (808 nm), nearly 85.55 ± 1.07% of the cells were killed, while in the absence of laser irradiation, the cell viability was approximately 100%. Local hyperthermia promotes the rupture of SIA‐*α*TSLs and the release of the chemotherapeutic drug Abemaciclib. Next, the in vitro cytotoxicity of Abemaciclib, A‐*α*TSLs, SIA‐*α*TSLs, and SIA‐*α*TSLs+L in CT26 cells were evaluated, respectively. In CT26 cells, all groups showed Abemaciclib dose‐dependent toxicity (Figure 3e). SIA‐*α*TSLs+L showed a better antitumor effect in vitro, with an IC_50_ value of 0.18 ± 0.10 µg mL^−1^, which was significantly lower than that of SIA‐*α*TSLs (*p* < 0.01) (Table S3, Supporting Information). Subsequently, the effects of SIA‐*α*TSLs on apoptosis and cell cycle arrest were examined. Upon flow cytometric analysis (Figure 3f, h), it was found that the rate of cell apoptosis in SIA‐*α*TSLs+L group increased to 40.50 ± 5.67%, while it was only 6.21 ± 2.21% in the control group. In Abemaciclib group, the proportion of cells in the G1 phase increased to 39.73 ± 2.89%, against only 29.13 ± 12.22% in the control group. The highest cell cycle arrest 49.42 ± 2.65% was in the SIA‐TSLs+L group (Figure 3g, i). These results indicated that SIA‐*α*TSLs have superior antitumor activity in vitro.

Studies have shown that local hyperthermia can cause tumor cells heat stress response and ICD.^[^ ^27^ ^]^ Heat shock protein 70 (HSP70) on the surface of tumor cells was detected after laser irradiation. The results showed that the expression of HSP70 after laser irradiation increased significantly (Figure 3j). Subsequently, we detected the expression of CRT, release of HMGB1, and secretion of ATP in CT26 cells, to study the ICD effect induced by SIA‐*α*TSLs. Fluorescence microscopy images and flow cytometric analysis revealed that after laser irradiation, the expression of CRT in SI‐*α*TSLs+L group was 3.18‐fold higher than that in SI‐*α*TSLs group (*p* < 0.001), while the expression of CRT in SIA‐*α*TSLs+L group was 1.62‐fold higher than that in SIA‐*α*TSLs group (*p* < 0.001) (Figure 3j, Figure S12, Supporting Information). The release of HMGB1 also showed similar results, SIA‐*α*TSLs+L group showed the highest release of 25.97 ± 3.25% (Figure S12, Supporting Information). Laser irradiation also significantly promoted the secretion of ATP, the secretion of ATP in SIA‐*α*TSLs+L group increased to 39.11 ± 0.93 nm, which was 5.3‐fold higher than that in SIA‐*α*TSLs group (Figure 3k). These results indicated that the local hyperthermia caused by laser irradiation could induce the ICD effect.

### 2.4 Antitumor Immune Responses of SIA‐*α*TSLs

**Figure 4:**
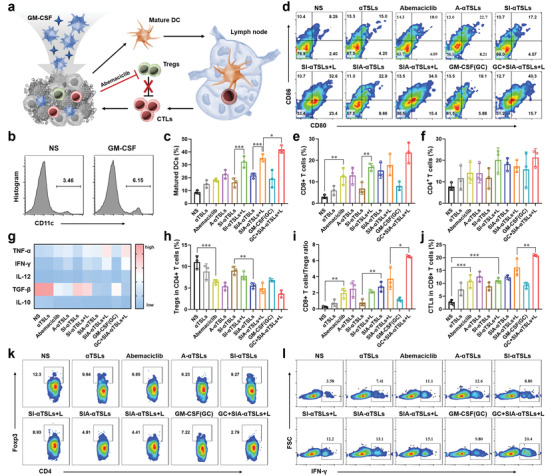
SIA‐*α*TSLs combined with GM‐CSF initiated antitumor immunity and enhanced the immune response. a) Schematic illustration of GM‐CSF recruiting the infiltration of DCs and Abemaciclib inhibiting the proliferation of Tregs. b) The proportion of DCs in the tumor tissue upon injection of NS or GM‐CSF. c,d) Flow cytometry analysis of DC maturation in lymph nodes (gated on CD11c^+^ cells). e,f) Intratumor infiltration of CD4^+^ and CD8^+^ T cells (gated on CD3^+^ T cells). g) The levels of intratumor cytokines, including TNF‐*α*, IFN‐*γ*, IL‐12, TGF‐*β*, and IL‐10, were quantified using ELISA. h,k) Percentage of Tregs in CD4^+^ T cells. i) Ratio of CD8^+^ T cells to Tregs. j,l) Percentage of CTLs in CD8^+^ T cells. Data were shown as mean ± SD (n  = 3). *p < 0.05, **p < 0.01, and ***p < 0.001.

DCs are the most effective antigen‐presenting cells, which can uptake, process, and present antigens to trigger an effective antitumor T cell response.^[^ ^28^ ^]^ Enhancing the infiltration of DCs into tumor tissues is essential for initiating antitumor immunity.^[^ ^29^ ^]^ MRI/NIRFI dual‐mode positioning‐guided PTT could promote the release of tumor‐associated antigens, following which GM‐CSF (GC) was injected into the tumor tissue to recruit the DCs to present the antigen. The results showed that after intratumoral injection of GM‐CSF, CD11c^+^ cells increased by 6.39 ± 0.70%, compared to those in the NS group (*p* < 0.01) (**Figure** **4**b, Figure S13, Supporting Information). DCs mature after ingesting the antigen and migrate to the lymph nodes. We detected mature DCs (CD11c^+^ CD80^+^ CD86^+^) in the lymph nodes. Flow cytometry results showed that the proportion of mature DCs in SI‐*α*TSLs+L group was higher than that in SI‐*α*TSLs group (*p* < 0.001), while the proportion of mature DCs in SIA‐*α*TSLs+L group was greater than that in SIA‐*α*TSLs group (*p* < 0.001), thus indicating that the PTT‐induced ICD effect could promote DCs maturation (Figure 4c, d). The proportion of mature DCs in GC+SIA‐*α*TSLs+L group was 41.93 ± 3.00%, which was significantly higher than that in SIA‐*α*TSLs+L group (*p* < 0.05), indicating that intratumoral injection of GM‐CSF could recruit DCs to take up antigens and promote the migration of mature DCs to lymph nodes.

Mature DCs present antigens to T cells to promote their activation and proliferation, following which the activated T cells migrate to tumor tissues to exert an immune‐killing effect. The T cells infiltrating the tumor tissue were separated and analyzed, and the results showed that GC+SIA‐*α*TSLs+L group had the highest proportion of CD8^+^ T cells and CD4^+^ T cells, which were 23.60 ± 4.81% and 21.33 ± 4.12%, respectively (Figure 4e, f, Figure S14, Supporting Information). CTLs that infiltrate tumor tissues are the main force for tumor killing, however, Tregs in tumor tissues inhibit their activity. Abemaciclib has been reported to selectively inhibit Tregs proliferation. We analyzed the proportion of Tregs in tumor tissues and found that the proportion of Tregs in the Abemaciclib, SIA‐*α*TSLs, and SIA‐*α*TSLs+L groups was significantly lower than that in NS, SI‐*α*TSLs, and SI‐*α*TSLs+L groups (*p* < 0.001, *p* < 0.01, *p* < 0.01) (Figure 4h, k). The GC+SIA‐*α*TSLs+L group showed the lowest proportion of Tregs at 3.63 ± 0.82% and the highest ratio of CD8^+^ T cells/Tregs at 6.52 ± 0.15% (Figure 4h, i). These results indicated that the proportion of Tregs was effectively reduced in SIA‐*α*TSLs group through the action of Abemaciclib. Subsequently, the proportion of CTLs infiltrating tumor tissue was analyzed. The proportions of CTLs in SIA‐*α*TSLs, SIA‐*α*TSLs+L and GC+SIA‐*α*TSLs+L groups increased to 12.70 ± 1.15%, 16.30 ± 4.52%, and 20.37 ± 0.55%, respectively (Figure 4j, l). This was accompanied by an increase in immune activation cytokine levels and a decrease in immunosuppressive cytokine levels (Figure 4g). These results indicated that GC+SIA‐*α*TSLs+L treatment could trigger an effective antitumor immune response.

### 2.5 In Vivo Antitumor Efficacy of SIA‐*α*TSLs

**Figure 5:**
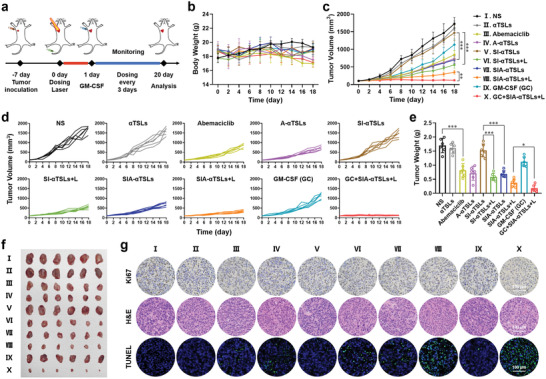
SIA‐*α*TSLs combined with GM‐CSF exhibited enhanced antitumor efficiency in CT26 tumor‐bearing mice. a) Schedule of in vivo administration approach. The mice were treated with different formulations and some groups were irradiated with 808 nm laser of 1.0 W cm^−2^ for 5 min. b) Body weight, c) average tumor growth curves, and d) individual tumor growth curves after intravenous injection with NS, *α*TSLs, Abemaciclib, A‐*α*TSLs, SI‐*α*TSLs, SI‐*α*TSLs+L, SIA‐*α*TSLs, SIA‐*α*TSLs+L, GM‐CSF(GC), and GC+SIA‐*α*TSLs+L. “S” represents SPIONs, “I” represents IR780, “A” represents Abemaciclib. e) Tumor weight and f) tumor photos at the endpoint. Data were shown as mean ± SD (n  = 6). *p < 0.05, **p < 0.01, and ***p < 0.001. g) Immunohistochemical images of the tumor tissues.

The antitumor effects of SIA‐*α*TSLs were assessed in CT26 tumor‐bearing BALB/c mice. The mice were divided into 10 groups and administered NS, *α*TSLs, Abemaciclib, A‐*α*TSLs, SI‐*α*TSLs, SI‐*α*TSLs+L, SIA‐*α*TSLs, SIA‐*α*TSLs+L, GM‐CSF (GC), or GC+SIA‐*α*TSLs+L. The administration schedule is listed in **Figure** **5**a. With the guidance of dual‐mode positioning, the tumor site was treated with laser radiation to kill tumor cells and induce ICD. Subsequently, GM‐CSF was intratumorally injected to recruit DCs to initiate antitumor immunity. The hemolysis rates of SIA‐*α*TSL at ≈10–500 µg mL^−1^ were negligible (< 5%), indicating that SIA‐*α*TSLs had good hemocompatibility and were suitable for intravenous administration (Figure S15, Supporting Information). During the experiment, the body weight and tumor volume were recorded every two days. In every group, the body weight of mice did not change significantly (*p* > 0.05), indicating that SIA‐*α*TSLs had lower systemic toxicity (Figure 5b). The tumor volume in SI‐*α*TSLs+L group was smaller than that in SI‐*α*TSLs group, indicating the effectiveness of PTT (*p* < 0.001). The tumor volume in SIA‐*α*TSLs group was smaller than that in SI‐*α*TSLs group, indicating the effectiveness of Abemaciclib (*p* < 0.001). The tumor inhibition rate of SIA‐*α*TSLs+L was 79.81 ± 5.30%, which was significantly higher than that of SIA‐TSLs+L group 72.28 ± 6.31% (*p* < 0.05), indicating that the aggregation of SIA‐*α*TSLs enhanced antitumor efficiency (Figure S16, Supporting Information). The GC+SIA‐*α*TSLs+L group displayed the best therapeutic effect and the tumor inhibition rate increased to 92.88 ± 0.84%, which was significantly higher than that in SIA‐*α*TSLs+L group (*p* < 0.01, *p* < 0.05) (Table S4, Supporting Information), indicating that the recruitment of DCs to initiate antitumor immunity could effectively enhance the effect of cancer therapy. After 20 days of treatment, the mice were euthanized and dissected, and the tumor tissues were collected for analysis. The weight of the isolated tumor and the tumor images were consistent with the results of the tumor volume (Figure 5e, f). Subsequently, the tumor tissues were analyzed using Ki‐67 immunohistochemistry, hematoxylin and eosin (H&E) staining, and TUNEL immunofluorescence to investigate the proliferation and apoptosis of tumor cells. The results showed that GC+SIA‐*α*TSLs+L group had the best ability to resist tumor proliferation and promote tumor apoptosis (Figure 5g). The heart, liver, spleen, kidney, and lungs were collected for H&E analysis. No obvious lesions were found in any of these groups, indicating that SIA‐*α*TSLs had no toxic effects on normal mouse tissues (Figure S17, Supporting Information). These results indicated that SIA‐*α*TSLs have superior antitumor effects and low systemic toxicity.

### 2.6 Long‐Term Immune Memory Effect of SIA‐*α*TSLs

**Figure 6:**
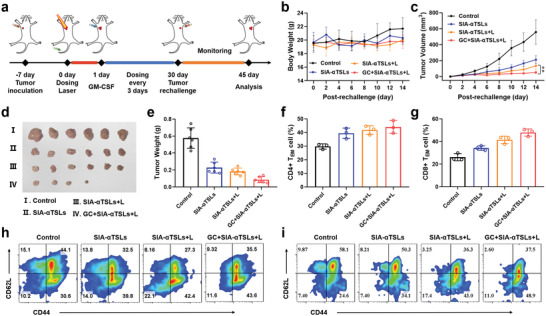
SIA‐*α*TSLs combined with GM‐CSF induced long‐term immune memory in CT26 tumor‐bearing mice. a) Schedule of in vivo administration approach and secondary tumor model establishment. b) Body weight of tumor‐bearing mice post‐rechallenge. c) The rechallenged tumor growth curves of tumor‐bearing mice treated with SIA‐*α*TSLs, SIA‐*α*TSLs+L, and GC+SIA‐*α*TSLs+L. d) Secondary tumor weight and e) tumor photos at the endpoint. Data were shown as mean ± SD (n  = 6). **p < 0.01. f,h) The frequency of CD4^+^ T_EM_ cells in spleens. g,i) Frequency of CD8^+^ T_EM_ cells in spleens. (gated on CD3^+^ T cells). Data were shown as mean ± SD (n  = 3).

Furthermore, we explored the potential of dual‐mode positioning therapy to prevent tumor recurrence. CT26 tumor cells were re‐inoculated into tumor‐bearing mice that survived after treatment to establish a secondary tumor model (**Figure** **6**a). In the control group, untreated BALB/c mice were inoculated with CT26 tumor cells. Body weight and tumor volume were measured every two days after re‐inoculation with tumor cells. During the rechallenge treatment, the body weight of mice did not change significantly (*p* > 0.05) (Figure 6b). The results of the re‐challenge treatment showed that the growth of secondary tumors was significantly delayed in mice pretreated with GC+SIA‐*α*TSLs+L, with the tumor completely eradicated in 2 mice (Figure 6c–e). The SIA‐TSLs and SIA‐TSLs+L groups also showed a delay in tumor growth. In contrast, the CT26 secondary tumors grew rapidly in the control group. These results indicated that GC+SIA‐*α*TSLs+L elicited a long‐term antitumor response in mice.

Effector memory T cells (T_EM_) play a crucial role in preventing tumor recurrence. After 15 d of rechallenge, we analyzed the proportion of T_EM_ in the spleen. The results showed that the proportion of T_EM_ in GC+SIA‐*α*TSLs+L group was higher than that in control group. CD4^+^ T_EM_ increased to 44.03 ± 4.67%, which was 1.48‐fold higher than that in control group (*p* < 0.01), while CD8^+^ T_EM_ increased to 47.93 ± 3.16%, which was 1.83‐fold higher than that in control group (*p* < 0.001). These results indicated that GC+SIA‐*α*TSLs+L could effectively stimulate antitumor immune responses and long‐term immune memory effects to prevent tumor recurrence.

## 3 Conclusion

In general, with the demand for clinical cancer PTT, we proposed a two‐wave variable nanotheranostic agent to enhance MRI/NIRFI dual‐mode imaging‐guided photo‐induced triple‐therapy for cancer. SIA‐*α*TSLs can induce cathepsin B‐responsive aggregation and NIR laser‐responsive dispersion, which could enhance MRI/NIRFI imaging and therapeutic effects. Under the guidance of MRI/NIRFI dual‐mode positioning, the tumor is irradiated with a NIR laser. First, local heating promotes the immunogenic death of tumor cells and released tumor‐associated antigens and DAMPs. Then, Abemaciclib was released after TSLs rupture, which leads to blocking of the tumor cell cycle and inhibition of the proliferation of Tregs. Third, intratumoral injection of GM‐CSF leads to recruitment of DCs and initiation of antitumor immunity. In CT‐26 tumor‐bearing mice, SIA‐*α*TSLs combined with GM‐CSF exhibited superior antitumor efficiency and induced long‐term immune memory. Collectively, this study provides a promising nanotheranostics strategy to improve cancer imaging and therapeutic efficacy.

## 4 Experimental Section

### Materials

Abemaciclib was purchased from MedChem Express Co. Ltd. (Shanghai, China). IR 780 was purchased from Aladdin Bio‐Chem Technology Co. Ltd. (Shanghai, China). DPPC was purchased from A.V.T. Pharmaceutical Co. Ltd. (Shanghai, China). DSPE‐PEG_2000_‐NHS (MW = 2978) was purchased from Xi'an Ruixi Biological Technology Co. Ltd. (Xi'an, China). CABT was purchased from Bide Pharmatech Co. Ltd. (Shanghai, China). Cathepsin B sensitive peptide [NH_2_‐AC(Trt)K(Boc)FR(Pbf)GD(tBu), MW = 1446] was purchased from Dangang Biological Co. Ltd. (Hangzhou, China). Antibodies for flow cytometry were purchased from BioLegend (San Diego, CA, USA). Cathepsin B and methylthiazol tetrazolium (MTT) were purchased from Sigma‐Aldrich (Shanghai, China). The cell cycle analysis, ATP assay, and Annexin V‐FITC/PI apoptosis detection kits were purchased from Beyotime Biotechnology Co. Ltd. (Shanghai, China). The ELISA kits were purchased from Dakewe Co. Ltd. (Shenzhen, China). All the reagents used were of analytical reagent grade.

### Cell Cultures

CT26 (mouse colon cancer cells) and HUVECs (human umbilical vein endothelial cells) were obtained from the Chinese Academy of Sciences. CT26 cells and HUVECs were cultured in RPMI‐1640 with streptomycin and penicillin (1%) and 10% (v/v) fetal bovine serum. Cells were cultured at 37 °C, in an incubator with 5% CO_2_.

### Animals

BALB/c mice (female, aged 6–8 weeks) were purchased from Beijing Vital River Laboratory Animal Technology Co. Ltd. (Beijing, China). Animal experiments were conducted according to the Guidelines for Care and Use of Laboratory Animals of Shandong University and were approved by the No.19030 Animal Ethics Committee of Shandong University.

### Synthesis of SPIONs

Ferric chloride hexahydrate and sodium oleate were dissolved in a mixed solution of 80 mL absolute ethanol, 140 mL n‐hexane, and 60 mL double distilled water, and heated at 70 °C for 4 h to obtain iron oleate. Iron oleate (40 mmol) and oleic acid (20 mmol) were dissolved in 1‐octadecene solution, and the reaction mixture was heated to 320 °C for 30 min to obtain black precipitates. The precipitates were washed with ethanol and dried to obtain SPIONs.

### Synthesis of Function Materials

DSPE‐PEG_2000_‐NHS and NH_2_‐AC(Trt)K(Boc)FR(Pbf)GD(tBu) were mixed in *N*,*N*‐Dimethylformamide (DMF) and stirred in the dark for 12 h. The product was stirred in a mixed solution (TFA:H_2_O:TIS = 95:2.5:2.5, v:v) at room temperature for 2 h and then placed in pre‐cooled ether to remove the protecting group. The precipitate was then centrifuged and washed with ether three times before vacuum‐drying to obtain the final product. The purity of the product was verified using ^1^H NMR. DSPE‐PEG_2000_‐NHS and CABT were dissolved in 1 mL of DMF. Both the solutions were then mixed and stirred in the dark for 24 h. The products were purified using a dialysis method, followed by lyophilization. The purity of the products was verified using ^1^H NMR spectroscopy.

### Preparation and Characterization of SIA‐*α*TSLs

SIA‐*α*TSLs were prepared using the film dispersion method. Briefly, DPPC, cholesterol, Abemaciclib, and IR780 were dissolved in a mixed solution of chloroform and methanol, evaporated at 40 °C to get a dry lipid film, followed by the addition of SPIONs with ultrasonic hydration for 10 min, to obtain SIA‐TSLs. DSPE‐PEG_2000_‐ACKFRGD and DSPE‐PEG_2000_‐CABT were then dissolved in 0.001 m PBS (pH = 7.4), added to the SIA‐TSLs, and incubated at 37 ℃ for 15 min to obtain SIA‐*α*TSLs. SIA‐*α*TSLs were extruded three times using membrane filters, and five times using LiposoFast‐Basic extruder. The morphology of SIA‐*α*TSLs was observed using TEM. The particle size, PDI, and zeta potential of SIA‐*α*TSLs were measured using Zetasizer Nano ZS90 (Malvern, Worcestershire, UK). Drug loading (DL%) and encapsulation efficiency (EE%) of IR780 and Abemaciclib in SIA‐*α*TSLs were measured using high‐performance liquid chromatography (HPLC) and UV–vis, respectively. The physical stability of SIA‐*α*TSLs was measured at 4 °C, and their size was measured every 3 d for 21 d.

### Characterization of Cathepsin B‐Responsive Aggregation

SIA‐*α*TSLs were placed in 1 mL of acetic acid‐sodium acetate buffer (pH = 5.0) and incubated with 2U of cathepsin B. SIA‐*α*TSLs without cathepsin B were used as the control group. Particle size changes of SIA‐*α*TSLs under different conditions were observed. The morphology of SIA‐*α*TSLs incubated with cathepsin B for 12 h was observed using TEM. Subsequently, the MRI and NIRFI imaging abilities of SIA‐*α*TSLs after aggregation were examined. Different concentrations of SIA‐*α*TSLs solutions with or without cathepsin B were subjected to MRI detection, and the transverse (T2) relaxation rates were acquired on a clinic 3.0 T MRI scanner (Siemens Prisma, Germany). The corresponding relaxation coefficients (r_2_) were calculated by linearly fitting the function of the inverse relaxation time to the metal‐ion concentration. Similarly treated SIA‐*α*TSLs were also measured using a real‐time IVIS spectrum system (Caliper Life Sciences, USA), the results were obtained by Living Image 3.1.

### In Vitro Photothermal Performance

The SIA‐*α*TSLs were irradiated using an 808 nm NIR laser at the powers of 0.1, 0.6, 1.0, and 2.0 W cm^−2^ for 10 min, to determine the optimal power and time. SIA‐*α*TSLs were dispersed in PBS at a series of concentrations and irradiated with a NIR laser at the power of 1.0 W cm^−2^ to examine the relationship between concentration and temperature. The temperature was recorded using a digital thermometer and an infrared imaging device. The morphology of SIA‐*α*TSLs after irradiation with 808 nm NIR laser was observed using TEM.

### NIR Laser‐Triggered Abemaciclib Release from SIA‐*α*TSLs

The release profile of Abemaciclib from SIA‐*α*TSLs and SIA‐*α*TSLs+L (1.0 W cm^−2^, 5 min) was measured using the dialysis bag method. SIA‐*α*TSLs were incubated in 10 mL PBS (pH = 7.4, containing 0.5% Tween‐80) at 37 °C (100 rpm). At predetermined time‐points, the release medium was replaced with a fresh medium. The concentration of released Abemaciclib was measured using HPLC. Each experimental group consisted of three replicates.

### Cellular Uptake In Vitro

Coumarin 6 (C6) was used as a tracer agent to obtain C6‐TSLs and C6‐*α*TSLs. HUVECs were incubated overnight in 12‐well plates (1×10^5^ cells/well). Fresh medium containing free C6, C6‐TSLs, and C6‐*α*TSLs was added to the wells and cultured for 1 and 4 h. HUVECs were pre‐incubated with free ACKFRGD (1 mg mL^−1^) for 1 h and then incubated with C6‐*α*TSLs for competitive inhibition experiments. Subsequently, the cells were washed with PBS and the nuclei were stained with DAPI. The fluorescence of cells was qualitatively observed using an inverted fluorescence microscope (Biotek, USA) and quantitatively determined using flow cytometry (CytoFLEX, Beckman, USA).

### MRI/NIRFI Imaging of SIA‐*α*TSLs In Vivo

The mice (CT26‐bearing) were divided into three groups, and injected with SIA‐TSLs, SIA‐A‐TSLs, or SIA‐*α*TSLs via the tail vein. Each mouse was scanned at four time‐points: before injection and 2, 6, and 12 h after injection. A 3.0 T MRI scanner (Siemens Prisma, Germany) was used for T2W1 imaging. The scanner was operated with a repetition time of 3500 ms, echo time of 113 ms, and layer thickness of 2.0 mm. A real‐time IVIS spectrum system (Caliper Life Sciences, USA) was used for in vivo NIRFI imaging. The major organs and tumors were excised for in vitro NIRFI imaging at 12 h after injection.

### In Vivo Photothermal Performance

The mice (CT26‐bearing) were divided into four groups, and were injected with NS, S‐*α*TSLs, I‐*α*TSLs, or SI‐*α*TSLs via the tail vein, respectively. 12 h after injection, the mice were anesthetized with 5% chloral hydrate via intraperitoneal injection and then the tumor tissue was irradiated with the 808 nm NIR laser at the power of 0.6, 1.0, and 2.0 W cm^−2^ for 10 min to determine the optimal power and time. Infrared thermal imaging was performed using an infrared camera at a pre‐determined time‐point.

### In Vitro Cytotoxicity

The cytotoxicity of SIA‐*α*TSLs in CT26 cells was assessed using an MTT assay. CT26 cells were incubated overnight in 96‐well plates (5×10^3^ cells/well). For PTT, different IR780 concentrations in the medium (0.001, 0.01, 0.1, 0.2, and 0.5 µg mL^−1^) were added and irradiated with an 808 nm NIR laser (1.0 W cm^−2^, 5 min). After adding MTT reagents, the cells were incubated for an additional 4 h and DMSO was added. Cell viability was measured using a microplate reader at 570 nm. Formulations containing different concentrations of Abemaciclib (0.01, 0.1, 1, 2, and 5 µg mL^−1^) were measured as described. Samples were measured in triplicate, and the data were shown as mean ± standard deviation (SD) (n  = 3). For cell apoptosis and cell cycle assays, cells were harvested and stained according to the manufacturer's instructions.

### In Vitro Immunogenic Cell Death Induction

CT26 cells were seeded into 24‐well plates (5×10^4^ cells/well) and incubated overnight. Cells were treated with NS, *α*TSLs, Abemaciclib, A‐*α*TSLs, SI‐*α*TSLs, SI‐*α*TSLs+L, SIA‐*α*TSLs, or SIA‐*α*TSLs+L. The laser conditions were 1.0 W cm^−2^ for 5 min. To detect the expression of HSP70, cells were labeled with the anti‐HSP70 primary antibody and AF594‐conjugated secondary antibody, and then observed with an inverted fluorescence microscope. To detect the exposure of CRT, cells were treated with 4% paraformaldehyde, labeled with anti‐CRT primary antibody and AF488‐conjugated secondary antibody, observed under an inverted fluorescence microscope, and quantified using flow cytometry. To detect the release of HMGB1, cells were treated (4% paraformaldehyde, 0.1% Triton X‐100), and labeled with anti‐HMGB1 primary antibody and secondary antibody (AF488‐conjugated), observed under an inverted fluorescence microscope, and quantified using ELISA assay kit. To detect the ATP secretion, the cell culture supernatant was collected after 12 h of incubation, and assayed using the ATP assay kit.

### In Vivo Immunization Study

The mice were treated with different formulations and some groups were irradiated with 808 nm laser of 1.0 W cm^−2^ for 5 min. Tumor tissues and lymph nodes were collected to prepare single‐cell suspensions using a copper mesh. Subsequently, cells were washed with PBS containing 1% rat serum. For DCs analysis, cells were stained with anti‐CD11c‐PE, anti‐CD80‐FITC, and anti‐CD86‐PerCP. For T cells analysis, cells were stained with anti‐CD3‐APC, anti‐CD4‐FITC, and anti‐CD8‐PE. For Tregs analysis, cells were stained with anti‐CD4‐FITC and anti‐Foxp3‐APC. For CTLs analysis, cells were stained with anti‐CD8‐PE and anti‐INF‐*γ*‐APC. The data for the samples were analyzed by FlowJo. ELISA assay kits were used to detect the secretion of TNF‐*α*, IFN‐*γ*, IL‐12, TGF‐*β*, and IL‐10 in tumor tissues according to the manufacturer's instructions.

### In Vivo Antitumor Activity

1×10^6^ CT26 cells were subcutaneously injected into the left axilla of BALB/c mice. The mice were randomly divided into 10 groups, when the tumor volume reached 100 mm^3^. The mice were then administered different formulations: I) NS, II) *α*TSLs, III) Abemaciclib, IV) A‐*α*TSLs V) SI‐*α*TSLs, VI) SI‐*α*TSLs+L, VII) SIA‐*α*TSLs, VIII) SIA‐*α*TSLs+L, IX) GM‐CSF(GC), and X) GC+SIA‐*α*TSLs+L. The laser conditions were 1.0 W cm^−2^ for 5 min. The dose of Abemaciclib, IR780, and SPIONs were 10.0, 1.0, and 1.0 mg kg^−1^, respectively. Body weight and tumor volume were measured every 2 days. Mice were sacrificed on the 20th day, following which the tumors and major organs were dissected, fixed with 4% paraformaldehyde, and if used for histological analysis, embedded in paraffin. The tumor sections were stained with H&E, Ki67, and TUNEL, major organs were stained with H&E.

### In Vitro Hemolysis Assay

The 2% red blood cells (RBCs) were isolated from rat blood and resuspended in NS. Then, 2.0 mL SIA‐*α*TSL (10, 50, 100, 250, and 500 µg mL^−1^) were incubated with 2.0 mL 2% RBCs at 37 °C for 3 h. Following the removal of RBCs from the suspensions through centrifugation (3000 rpm, 10 min), the samples were photographed and the absorbance of hemoglobin was measured by UV–vis at the wavelength of 576 nm.

### Long‐Term Immune Memory

BALB/c mice (CT26‐tumor‐bearing) were treated with SIA‐*α*TSLs, SIA‐*α*TSLs+L, and GC+SIA‐*α*TSLs+L. The surviving mice were re‐inoculated with 1×10^6^ CT26 cells in the right axilla, at 30 d post‐treatment to establish a secondary tumor model. In the control group, untreated BALB/c mice were inoculated with CT26 tumor cells. Body weight and tumor volume were measured every two days after re‐inoculation with tumor cells. The spleens from surviving mice were harvested and filtered through a copper mesh to obtain a single‐cell suspension. Cells were stained with anti‐CD3‐APC, anti‐CD4‐PE (or anti‐CD8‐PE), anti‐CD62L‐FITC, and anti‐CD44‐PerCP‐Cy5.5 to analyze CD4^+^ T_EM_ and CD8^+^ T_EM_.

### Statistical Analysis

One‐way analysis of variance (ANOVA) analysis was used to evaluate the statistical significances among different groups using Prism software (version 8.0, GraphPad). Statistical significance was presented as *p < 0.05, **p < 0.01, and ***p < 0.001. All data were extracted from at least three independent experiments and presented as the mean ± SD.

## Conflict of Interest

The authors declare no conflict of interest.

## Supporting information

Supporting Information

## Data Availability

The data that support the findings of this study are available from the corresponding author upon reasonable request.
