# Supplementary material for: Two‐Wave Variable Nanotheranostic Agents for Dual‐Mode Imaging‐Guided Photo‐Induced Triple‐Therapy for Cancer
Source: Adv Sci (Weinh). 2022 Aug 2;9(27):2201834. doi: 10.1002/advs.202201834 (PMC9507363; doi:10.1002/advs.202201834)
Supplement: Supplementary file 1 — Supporting Information [file ADVS-9-2201834-s001.pdf]

Supporting Information

Two-wave Variable Nanotheranostic Agents for Dual-mode Imaging-guided Photo-induced Triple-therapy for Cancer

Xiao Sang‡, Tong Gao‡, Xiaoqing Liu, Yelong Shen, Lili Chang, Shunli Fu, Han Yang, Huizhen Yang , Weiwei Mu, Shuang Liang, Zipeng Zhang, Na Zhang, and Yongjun Liu**

‡These authors contributed equally
*Corresponding Author

X. Sang, T. Gao, X. Liu, L. Chang, S. Fu, H. Yang, H. Z. Yang, W. Mu, S. Liang, Z. Zhang, Prof. N. Zhang, A. Prof. Y. Liu
Department of Pharmaceutics, Key Laboratory of Chemical Biology (Ministry of Education), NMPA Key Laboratory for Technology Research and Evaluation of Drug Products, School of Pharmaceutical Sciences, Cheeloo College of Medicine, Shandong University
44 Wenhua Xi Road, Jinan, Shandong Province 250012, China
E-mail: zhangnancy9@sdu.edu.cn; liuyongjun@sdu.edu.cn

Y. Shen
Department of Radiology, Shandong Provincial Hospital Affiliated to Shandong First Medical University
324 Jingwu Weiqi Road, Jinan, Shandong Province, 250021, China

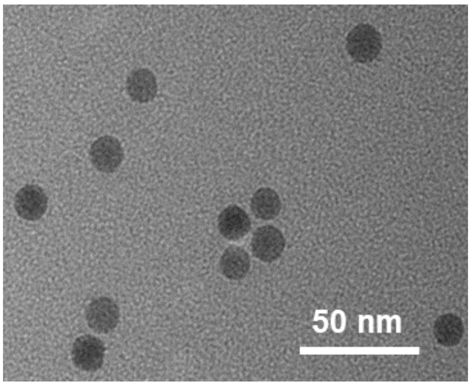

Figure S1. TEM image of SPIONs.

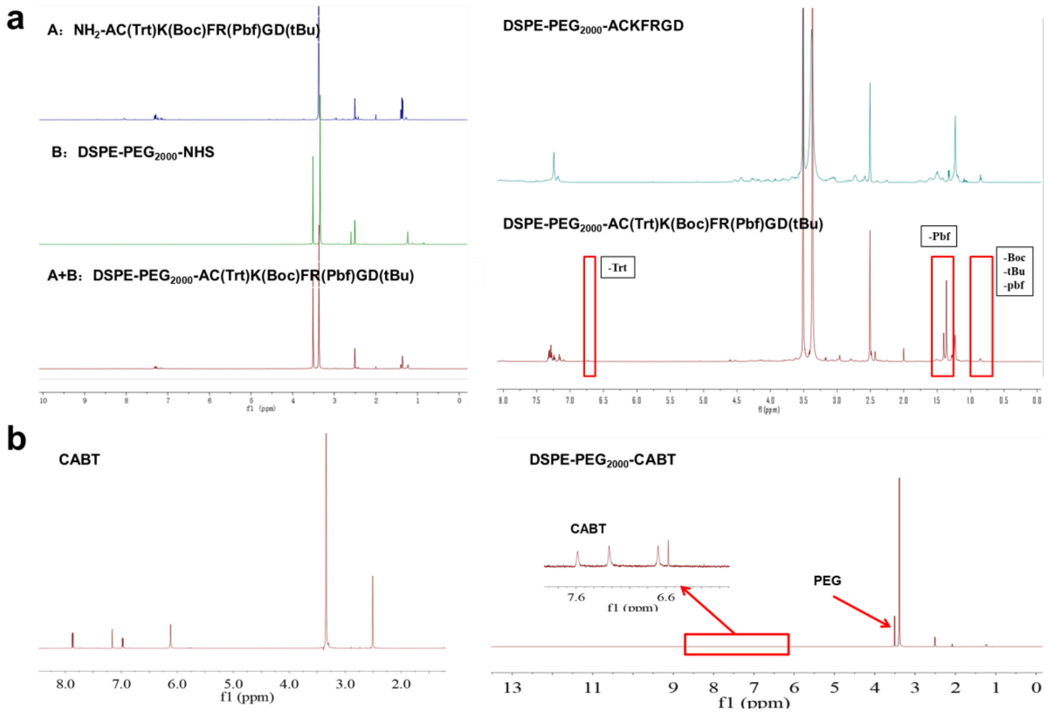

Figure S2. ^{1}H-NMR spectra of a) DSPE-PEG_{2000}-ACKFRGD and b) DSPE-PEG_{2000}-CABT.

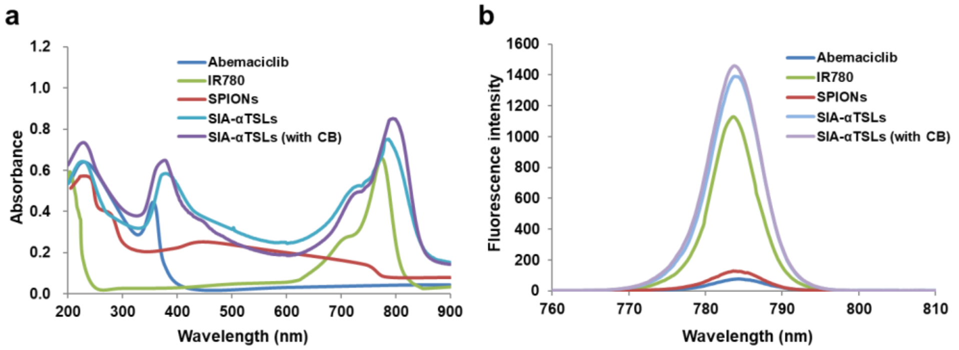

Figure S3 a) UV-Vis absorption spectra and b) fluorescence spectra of Abemaciclib, IR780, SPIONs, SIA-αTSLs and SIA-αTSL with cathepsin B.

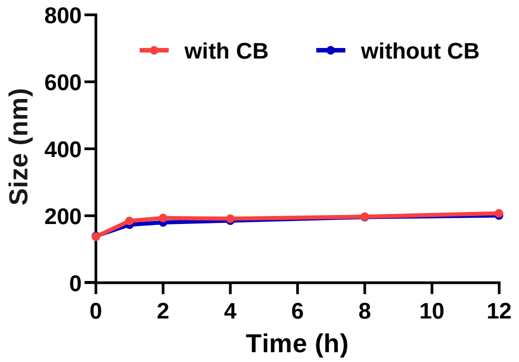

Figure S4. The size changes of SIA-TSLs incubated with or without cathepsin B.

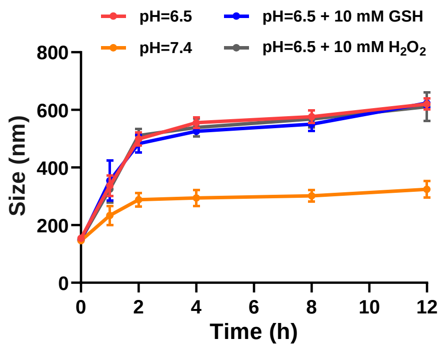

Figure S5. The size changes of SIA-αTSLs incubated with cathepsin B at different conditions.

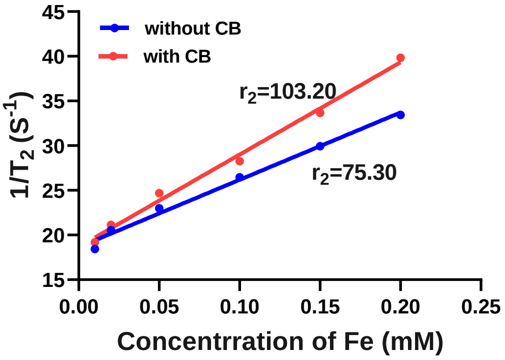

Figure S6. The transverse relaxation rate (r2) of SIA-TSLs incubated with or without cathepsin B.

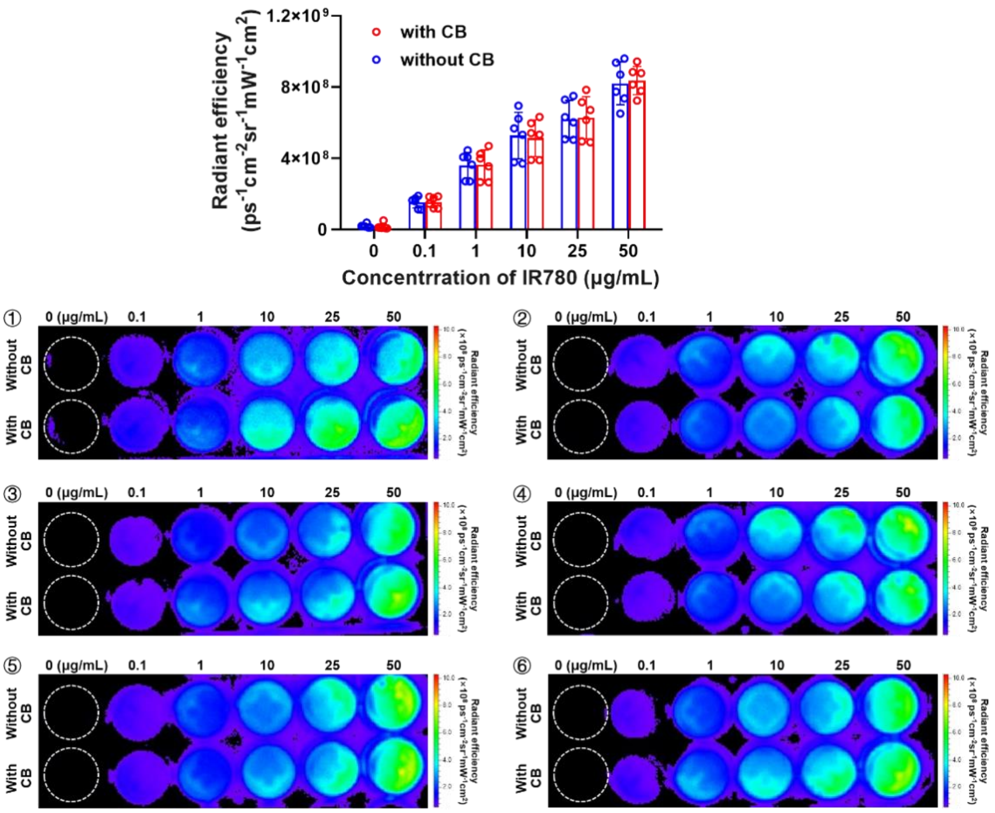

Figure S7. NIRFI fluorescence image and intensity quantification of SIA-αTSLs incubated with or without cathepsin B.

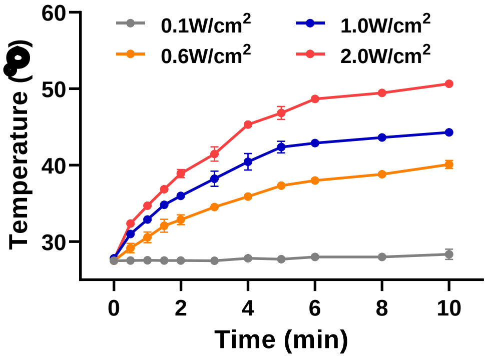

Figure S8. Temperature profile of SIA-αTSLs under laser irradiation at different powers.

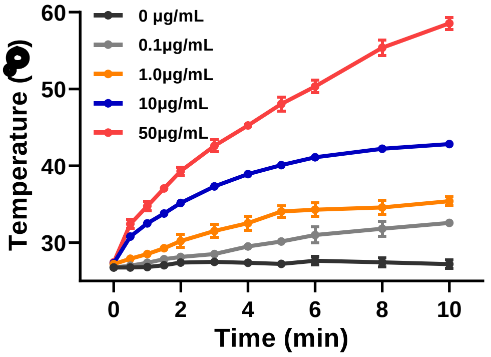

Figure S9. Temperature profile of SIA-αTSLs with different concentration under laser irradiation (808 nm) at 1.0 W/cm^2.

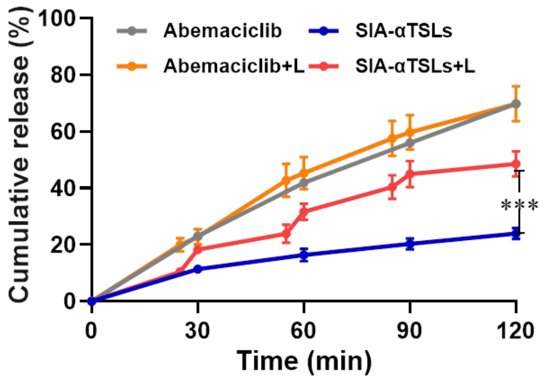

Figure S10. Release profiles of Abemaciclib from SIA-αTSLs within 2 h with or without laser irradiation. ***$p < 0.001$.

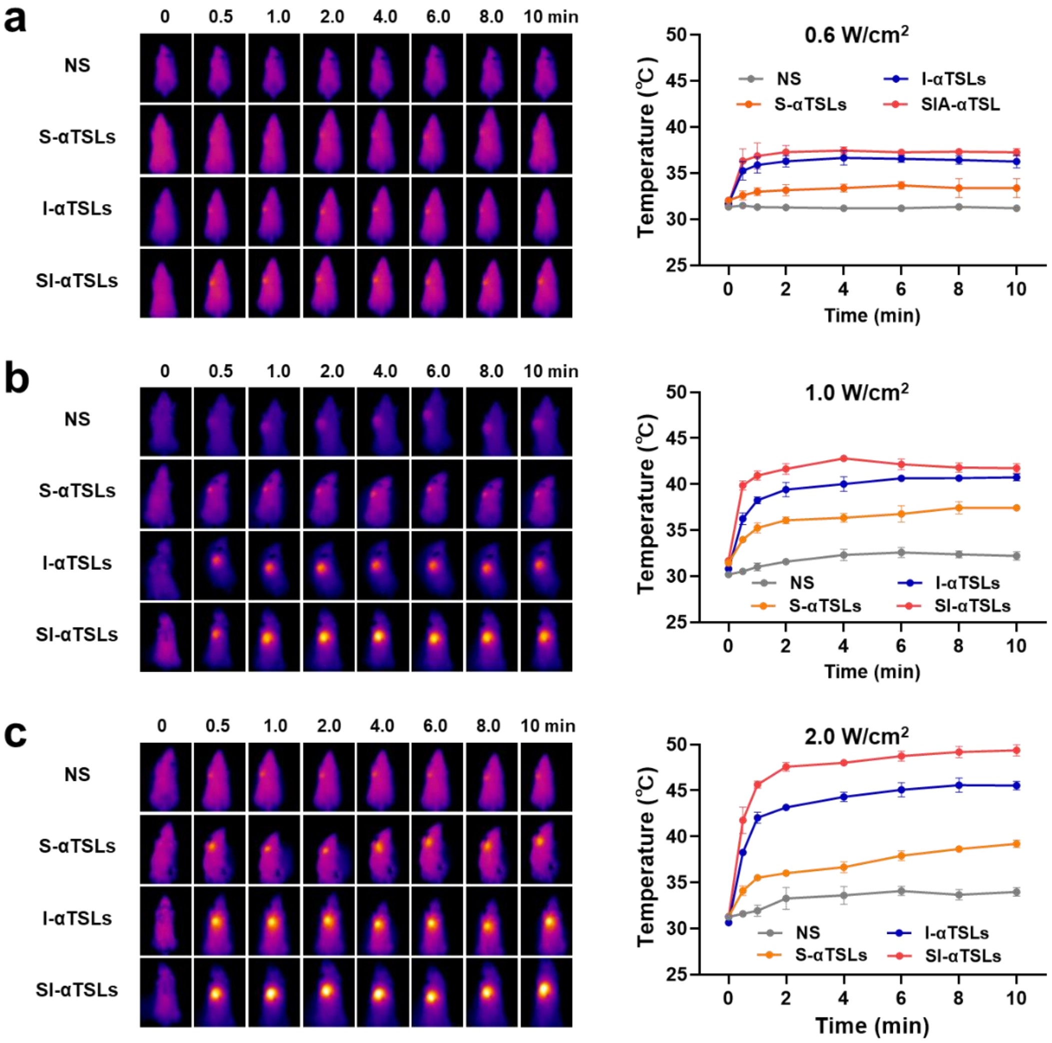

Figure S11. Infrared thermal images and temperature profile of tumors within 10 min under a) 0.6 W/cm^2 laser irradiation, b) 1.0 W/cm^2 laser irradiation and c) 2.0 W/cm^2 laser irradiation.

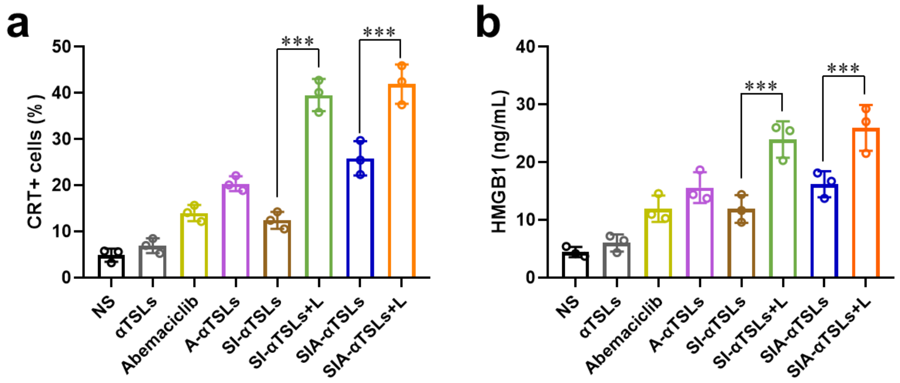

Figure S12. a) Flow cytometric analysis of CRT exposure on CT26 cells. b) ELISA analysis of HMGB1 released from CT26 cells. ***$p < 0.001$.

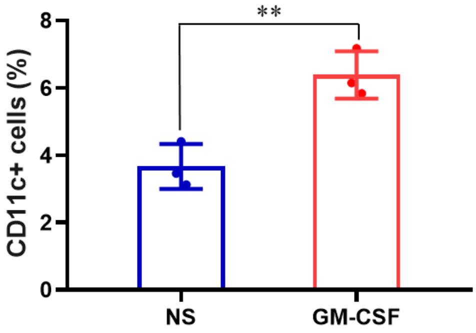

Figure S13. Flow cytometry analysis of $CD11c^+$ cells in tumor tissue after intratumoral injection of NS or GM-CSF. $^{**}p < 0.01$.

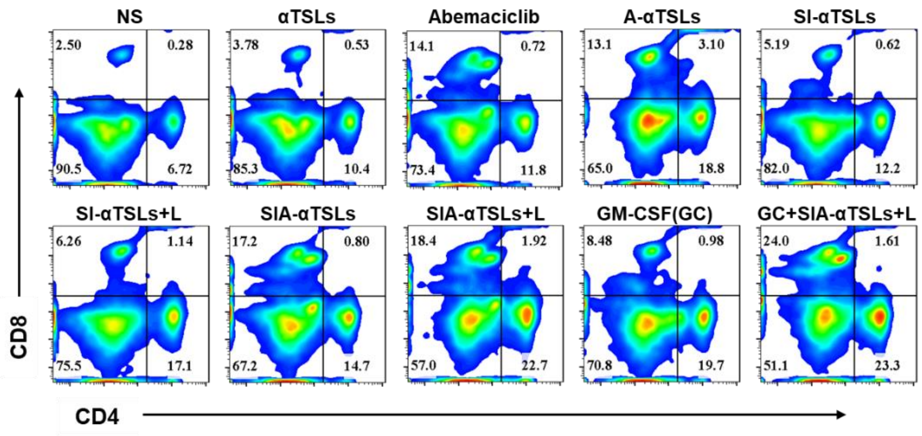

Figure S14. Flow cytometry analysis of $CD4^+$ and $CD8^+$ T cells in tumor tissue (gated on $CD3^+$ T cells).

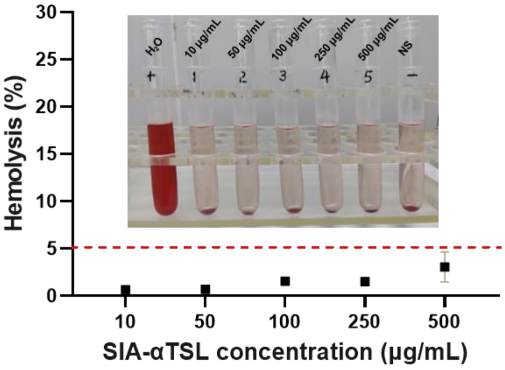

Figure S15. Hemolysis percentage of RBCs and the images after RBCs suspension was incubated with SIA-αTSL.

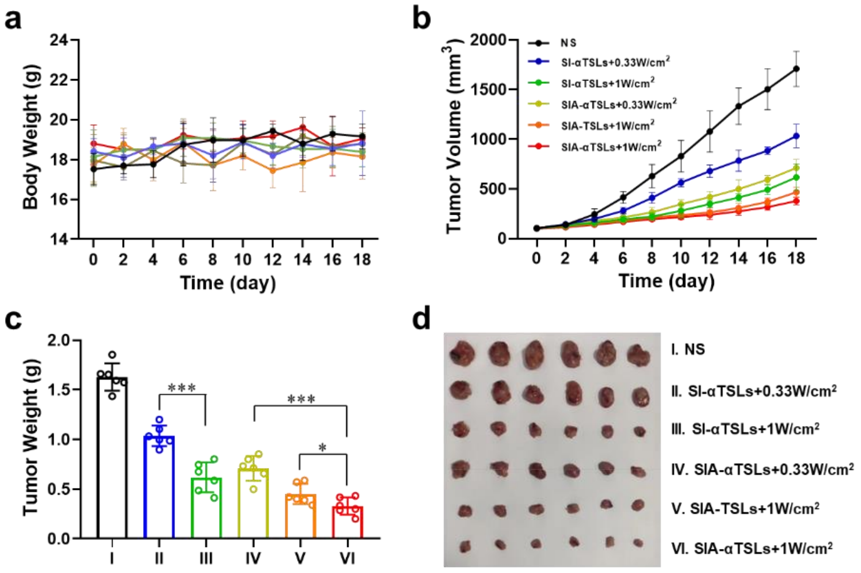

Figure S16. a) Body weight b) average tumor growth curves of mice after intravenous injection with different formulations. c) Tumor weight and d) tumor photos at the endpoint. “S” represents SPIONs, “I” represents IR780, “A” represents Abemaciclib, “α” represents that the TSLs can aggregate. Data were shown as mean ± SD (n = 6). **p* < 0.05 and ****p* < 0.001.

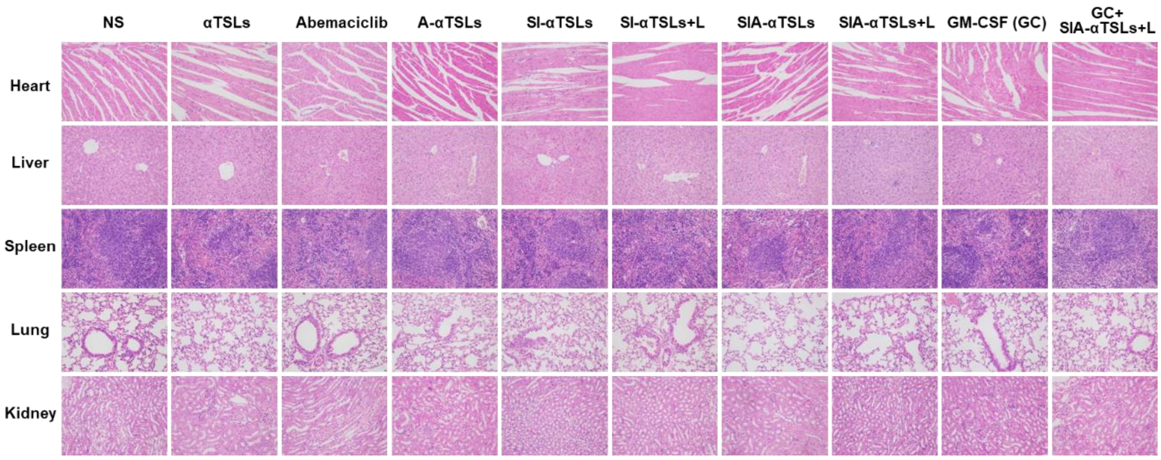

Figure S17. Immunohistochemical analysis. Representative microscopy images of H&E-stained histological sections of CT26 tumor-bearing mice after treated with different formulations. Magnification: 200 ×.

Table S1. Characterization of TSLs, SIA-TSLs, SIA-A-TSLs and SIA-αTSLs. Data were shown as mean ± SD (n=3).

Groups	Size (nm)	PDI	Zeta (mV)
TSLs	101.80 ± 3.44	0.105 ± 0.015	-1.30 ± 0.26
SIA-TSLs	139.57 ± 4.27	0.145 ± 0.049	-6.16 ± 0.54
SIA-A-TSLs	146.30 ± 3.80	0.168 ± 0.051	-6.85 ± 0.90

SIA-αTSLs	148.13 ± 4.24	0.120 ± 0.052	-4.69 ± 0.17

Table S2. Drug loading efficiency and encapsulation efficiency of Abemaciclib, IR780 and SPIONs in SIA-αTSLs. Data were shown as mean ± SD (n=3).

Drugs	Drug loading (%)	Encapsulation efficiency (%)
Abemaciclib	9.87 ± 0.22	95.34 ± 2.19
IR780	0.90 ± 0.04	88.50 ± 0.92
SPIONs	0.80 ± 0.05	40.12 ± 2.77

Table S3. The IC_{50} values of Abemaciclib, A-αTSLs, SIA-αTSLs and SIA-αTSLs+L in CT26 cells. Data were shown as mean ± SD (n=3). $^{**}p < 0.01$, compare with SIA-αTSLs group.

Groups	IC_{50} (μg/mL)
Abemaciclib	2.71 ±0.70
A-αTSLs	2.10 ± 0.52
SIA-αTSLs	1.81 ± 0.12
SIA-αTSLs+L	0.18 ± 0.10 **

Table S4. Tumor inhibition rate of tumor-bearing mice after intravenous injection with different groups. Data were shown as mean ± SD (n=6).

Groups	Tumor inhibition rate (%)
αTSLs	7.04 ± 11.88
Abemaciclib	50.87 ± 7.33
A-αTSLs	57.35 ± 5.57
SI-αTSLs	14.88 ± 12.90
SI-αTSLs+L	67.55 ± 5.46
SIA-αTSLs	59.27 ± 6.18
SIA-αTSLs+L	79.71 ± 3.27
GM-CSF(GC)	34.25 ± 9.39
GC+SIA-αTSLs+L	92.88 ± 0.84
